# Outlines of Military Surgery

**Published:** 1839-04-01

**Authors:** 


OUTLINES OF MILITARY SURGERY. By Sir *George Ballingall*, M.D. F.R.S. &c. Regius Professor of Military Surgery in the University of Edinburgh. Second Edition. Longman and Co. pp. 538.

How much the science of medicine owes to the labours and the contributions of naval and military surgeons would form neither an uninteresting nor unfruitful inquiry. More we are inclined to believe might be traced to these sources than is generally yielded to them. From the days of Paré, whose name would have been immortalised in the annals of the healing art, by one improvement had he done nothing more—the invention or revival—and how nearly synonymous these terms become—of the ligature of arteries in amputations and wounds:—from Paré down to the distinguished surgeons both Foreign and English of the last war, how numerous and how valuable the improvements, modifications, and discoveries which they have bequeathed, not to all succeeding military and naval surgeons only—but to medical science in its largest limits.

By tracing the progress of the healing art during the last two hundred years, this might be easily demonstrated and the names of surgeons of both services attached to those epochs in surgery, formed not by time, but by the improvements and advances which have most contributed to place the science in the rank it holds at present. It will, however, be more useful perhaps to show upon what these good results, which cannot be seriously disputed depend, and how far governments, and more particularly our own, have adopted the measures best calculated to draw the utmost fruit from such valuable sources. Although a long peace, which fortunately all countries have rather sought to maintain than disturb, has rendered such a subject less immediately interesting—yet it is in peace that we should prepare for war, and the elements of discord in the political atmosphere of the present day are sufficiently rife to lead us into war at no distant period, in spite of our debt which is real—or the amity of France and the United States—or the neutrality of Russia—all of which seem more or less apocryphal.

The slightest inquiry into the causes of the many and valuable improvements and contributions to science derived from the medical officers of armies and navies, leads us at once to the circumstances on which they depend and the chief of these is well laid down in a single paragraph.

"It is not, however, in a combination of the two great branches of the profession that the essence of Military Surgery consists, so much as in the peculiarity of the circumstances in which they fall to be exercised; and it is not perhaps more in the surgical than in the medical division of the subject that this peculiarity exists. Military Surgery may be considered as a judicious application of all the subdivisions of the healing art to those varied circumstances in which soldiers are placed both in health and in sickness. 'Ce n'est pas toutefois une science distincte, ni même une branche particulière de l'art de guérir, mais une application raisonnée de toutes les parties de cet art aux circonstances variées dans lesquelles se trouvent les hommes de guerre, tant en santé qu'en maladie.'" 2.

The necessity for ample knowledge and the daily experience in all the branches of the healing art have a constant tendency to enlarge the scope of the surgeon's observations, and above all, to prevent narrow or contracted views of diseased actions and their remedies. The division of labour and distinctions into classes which obtain in cities, may have some good results in *practice*, but unfortunately these distinctions which ought only to commence there, sometimes are observable ab ovo—exercising a most pernicious influence through the student's education, where he has decided from the beginning that he will be a physician, a surgeon, an accoucheur, or an oculist only, leading to the acquirement of a partial and very imperfect knowledge of the science generally, which becomes daily more contracted by the limited class of cases to which the attention is confined.

In this we hold the medical officers of the public service have at all times been placed in circumstances conferring great and important advantages.

A second class of advantages, not less notable, consists in the extensive nature of the experience acquired—thus

"All arts, trades, and professions, expose those who practise them to different diseases, the number, nature, and severity of which vary, according to the influences, more or less hurtful, to which individuals are subjected, and according to the kind of life which, by custom or necessity, they are compelled to lead. While many of the exercises and habits of soldiers are undoubtedly of an invigorating and salutary description, there is no class of men exposed to more numerous or more fertile sources of disease, in the frequent, sudden, and unexpected changes of climate to which they are subjected,—in the urgent and unseasonable calls of duty which they must not disobey,—in the privation of food, of shelter, and of every comfort to which they must occasionally submit; and in the thoughtless excesses of every kind in which they are too prone to indulge. If to the study of all these causes, and their effects upon the human frame, we add the consideration of those complicated wounds which it is their peculiar lot to receive, we shall find abundant occupation for the exercise of all the talent and industry which the most active and assiduous professional man can bring to bear upon the subject of military medicine and surgery. 'All those who have practised medicine in armies have observed, that the practice of it there presents difficulties which are very remarkable, whether on account of the nature and severity of the affections which attack soldiers, on account of the complicated forms in which those affections are met with in camps and in hospitals, or on account of numerous obstacles and interruptions to the usual and approved modes of treatment in civil life. Hence it is that, independent of the theoretical and practical knowledge necessary for every physician, he who is destined for the service of the army ought to have accurate and precise information with regard to the condition of the soldier, his manner of life, his habits, his duties, his exercises, the numerous causes of disease which beset him, and the extraordinary circumstances in which he is placed, particularly in the time of war.' " 5.

Without following the subject further, this glance will be sufficient to show the great advantages constantly attendant on military practice. The arguments quoted are brought forward by Sir George, not to prove the position we have chosen, but the necessity for previous instruction in military surgery, and the establishment of schools or lectures for that purpose, not only in Edinburgh but in London and Dublin, for the due preparation of those who devote themselves to either service. When we remember the class of youths of 17 and 18, half educated and totally ignorant of all circumstances connected with the diseases and accidents of soldiers and sailors, who filled the junior ranks in both services during the late war, it cannot but appear highly desirable that some efficient measures should be taken, to insure at any time the proper, and in some degree particular, instruction, required to enable a surgeon to perform all his duties efficiently, in either service.

At the present moment such is the overplus of candidates for professional employment, that doubtless, were there a war now, the first or second year's levies and operations might be readily and abundantly supplied with fairly educated young men, but even these would be totally unprepared for the thousand exigencies and peculiarities of service whether by sea or land —and after that period our armies and our fleet would be in much the same condition as they were 40 or 50 years ago!

The position of the junior medical officers in both services, and more particularly in the navy, is not such as to offer any inducement to well-educated men, who have any prospects, however mediocre, in civil life.

Much has been effected since the beginning of the last war—the great injustice of the then existing regulations and that which has more weight with governments—the inevitable and lamentable consequences of treating those to whom the lives and limbs of their soldiers or sailors were confided, with scanty remuneration, and less consideration, became so evident, that gradual and important ameliorations have since that period been effected. Much, however, is yet to be done in the English service before the medical officers are placed in the position to which their education, zeal, and the nature of their services have ever entitled them—and let us add, before the same inducements which are held out to their brother officers, for the highest exertion and the cheerful devotion of their energies under any accumulation of trying and perilous circumstances, shall have also been afforded to the medical staff. Perhaps have governments, not less than generals, been but imperfectly aware, of how much the success of their armies, on which they lavish millions of money, and of their generals, on whom dukedoms, stars, and wealth are showered, depends upon their sick list—upon the sanitory state of their armies—and these upon the efficiency and devotion of the medical officers. Perhaps too both the English government and the commanders of its fleets and armies, finding that their regulations, however unjust, or their modes of remuneration however scanty and relatively unfair, were still responded to by a general efficiency and many examples of high talent and devoted zeal, have formed their opinions from these results of a high feeling of that which medical officers felt they owed to themselves and their country,—and thus rested content midway. Be this as it may, much is yet to be done before the medical officers of the public services of Great

Britain are even placed in the same position as those of many other countries. And even in those where they are most favourably placed, it may be questioned whether all has been done which justice and the best interests of governments demand.

As Sir George himself remarks—

"Military Surgery, as it now exists, is so essentially the creation of the late war, its principles have been so fully developed, and its future practice must be so much influenced by the experience acquired in the recent campaigns, that to this experience I must chiefly refer in laying down the rules of treatment applicable to those formidable diseases which assail our fleets and our armies, and to those severe and complicated injuries which it is the lot of the soldier and the seaman to sustain." 6.

Much novel or original matter is not therefore to be looked for—the object of the work being rather to give a clear and succinct account of the various circumstances of military service—the diseases and injuries of soldiers as they had been ascertained and described at the close of the last war, with such plain and straightforward instructions as may be best calculated to fit a young surgeon for his military duties. It would be fruitless, therefore, to follow the author through his various chapters on gun-shot wounds, amputations, &c. We may observe, however, that in this second edition Sir George has given ample evidence of having carefully selected any original or valuable information which such additional experience as the few partial storms of European politics have furnished—Navarino—Antwerp—and the wars of succession in Portugal and Spain.

The sections devoted to the diseases of camps and garrisons of troops on foreign stations and the proportion of sick and wounded in armies, embrace subjects of interest at every time, and well merit consideration.

The medical statistics of armies have never yet been cultivated so as to give the varied and valuable information which they are well capable of doing under a better system. How important are the considerations and results involved in this subject, is well pointed out when quoting Sir James M'Grigor. The author shows—

"During the ten months from the siege of Burgos to the battle of Vittoria, inclusive, the total number of sick and wounded which passed through the hospitals was ninety-five thousand three hundred and forty-eight. By the unremitting exertions of Sir James M'Grigor and the medical staff under his orders, the army took the field preparatory to the battle with a sick-list under five thousand. For twenty successive days it marched towards the enemy, and in less than one month after it had defeated him, mustered within thirty men as strong as before the action,—and this too without reinforcements from England, the ranks having been recruited by convalescents." 62.

"But it is in the navy, even more than in the army, that the effects of prophylactic measures, both medical and military, have had the most conspicuous influence in diminishing sickness and mortality."

"A long sea voyage was formerly considered one of the most unhealthy situations to which a man could be exposed, while by the institution and enforcement of prophylactic measures a ship's company may now be conducted round the world, exposed to every vicissitude of temperature, and to all the hardships and dangers of the sea, with a smaller proportional loss of men than would happen in almost any other given situation." 63.

Few subjects of equal interest and weight have been more neglected, or perhaps, which is worse, more loosely and vaguely treated, than the medical statistics of armies, rich in facts, and promising, both in a political and scientific point of view, valuable results; it is a matter which has been left principally to the adjutant and serjeant-major, referring chiefly to the ranks absent or present; to some hospital returns in which numbers still are the object, but rarely or never combined in such order and system as to furnish all the data necessary to the development of certain important averages and general principles.

And how should they? since but little labour seems to have been bestowed to define what are the general results, the conclusions, and the principles which it is desirable to establish upon an unerring basis. These, it seems to us, are—

*First*—The causes affecting the health and lives of soldiers, classed in their relative value, as to frequency and fatality.

*Secondly*—These causes once clearly ascertained, defined, and classed, their mode of action, the precise degree and duration of their effects, would form the next subject of inquiry.

*Thirdly*—The various means of preventing, modifying, or controlling all injurious influences divided into—measures emanating from the commander-in-chief, and measures under the immediate coutrol of the medical staff—such as medicinal treatment, &c.

From such a series of data—independent of the first great advantage of passing regulations and taking measures best calculated to insure the least possible loss and suffering—would flow one of great political importance—viz. the power of *anticipating* the general results of warlike operations, and consequently determining the measures necessary to meet the waste of men—diminution of effective strength, hospital stores, &c.

Many returns have been published at different periods, but neither any one set, nor all put together will give the facts necessary, and for a simple reason; the true aim and objects of medical statistics, as applied to armies or fleets, have never been sufficiently well ascertained or clearly defined. Precise and abundant information bearing upon and elucidating certain points, can never be collected while those points are either not held in view or vaguely perceived. Sir George remarks on this subject, that—

"Some interesting and valuable statistical information upon this subject has lately been given to the public by Mr. Edmonds, and by Mr. Alcock, in his 'Notes on the Medical History and Statistics of the British Legion in Spain.' Some of the more important points on which his tables bear, are the ultimate loss to the effective strength of an army from wounds, and the scale, in which the first loss after an action progressively diminishes; the average mortality from musket wounds; the proportion which the different classes of wounds bear to each other; and this with reference to the wounded of a force attacking and defending batteries, houses, and lines, skirmishes and actions in the open field, and the assault of a fortified town." 63.

Neither of these gentlemen, however, has fulfilled all the indications, and it remains for those engaged in the next war to furnish the data so much required. They must first begin by defining all the principal points to be elucidated, for it is to the want of *previously formed* and clear conception of the whole scope and aim of the inquiry, that Mr. Alcock seems justly to attribute his being enabled only partially to carry out his views on this subject, by the facts he collected while with the armies of Spain and Portugal.

From the imperfect return already in existence, Mr. Alcock seems to have demonstrated two or three conclusions not hitherto generally received—conclusions and averages calculated to influence the operations and success of a war. Thus

"The period of smallest loss to an army is a victorious and vigorously prosecuted campaign, with frequent battles and much marching." 63.

That an inclement Winter, ushered in by a wet Autumn, *passed by troops in quiet cantonments*, is the most destructive period.

That the heat of Summer is as injurious as the rains or the cold of the succeeding seasons, causing as much sickness and mortality. Hence, in an active and successful campaign, different seasons produce nearly similar results.

To follow out the subject beyond these indications of the value and importance of the information wanted, would lead us much too far.

On hospital arrangements and the transport of wounded, the author has given some excellent observations in reference to two points of military organization, in which we are far inferior to some other armies, and notably to the French. The formation of an ambulance for the transport and immediate treatment of wounded, and an hospital corps for the due and efficient attendance on the sick in hospitals, &c. are indispensably necessary. Nothing can be more defective or pregnant with evil to the service, than the present regulations—injurious alike to the healthy and the non-effective. Sir George truly remarks—

"For this purpose, the only effectual provision seems to be, the formation of an Hospital corps, placed entirely at the disposal of the medical staff, and consisting of men either enlisted and embodied solely with this view, or transferred to the hospital establishment in consequence of having, from years or from accidents, become less effective in the line. A body of men of this description, trained to the particular duties required of them, qualified to attend the sick in the hospitals, as well as to succour and bear off the wounded in the field, would preserve the integrity and effective force of regiments; would afford a degree of comfort to the sick and wounded, to which they are too often strangers; and would give an efficiency to the medical staff, which the most zealous devotion to the duties of the service cannot otherwise ensure." 88.

This experiment was fully tried, it seems, in the Legion, and with the happiest results.

Our means of transport for the wounded are very ill adapted and arranged, and certainly call for no little reform. We have understood that, on these two heads, some measures were in contemplation by the government, at which we sincerely rejoice, for much are they required.

A large portion of the work—devoted to the consideration of wounds in all the variety of nature and circumstance attendant on a soldier's injuries—contains a clear and well-arranged digest of the present state of our knowledge on military surgery, with many original and interesting remarks and suggestions.

With some surprize we observe a practice we had long thought abandoned together with the charms and wonder-working salves of the dark ages, described as still prevalent in the French army.

"It would appear that this practice of indiscriminate dilatation is not yet abandoned by the French surgeons, for Mr. Alcock in a letter to me, states, that upon a very recent occasion, after the assault of Irun, a military ambulance was dispatched from Bayonne with offers of assistance to the wounded; and that all the Spanish who fell into their hands were unmercifully estrellated by the French bistoury. To this general rule there is, however, at least one creditable exception. M. Baudens, chief surgeon to the French troops serving at Algiers, seems to have had his eyes opened to the mischievous effects of this exclusive rule; and his conversion seems to me a point of much interest and importance." 216.

We may remark, *en passant*, that we were struck by the poverty of the leçons given by Dupuytren, on the gun-shot wounds resulting from the days of July, 1830, the first time the printed account of them met our eyes. They seem to be a pas en arrière to Larrey, instead of being, as was naturally to be expected, something in advance.

The *torsion* of arteries, now nearly totally relinquished, seems to have been extensively tried within the last few years.

"Torsion has been said to act less certainly on the smaller than on larger-sized arteries, and hence, perhaps, the little progress it has made in this country. Mr. Alcock tells me that torsion was tried in fourteen consecutive amputations, at Oporto, including one at the shoulder-joint, and that secondary hæmorrhage occurred only in one of them." 231.

As an outline of lectures forming a clear and tolerably concise digest of the principal subjects within the scope of military surgery, and of most importance to the young surgeon about to enter on such a career, it is an excellent work, and much care and labour in condensing old matter and adding new, has evidently been bestowed upon this edition, with great advantage to the reader.

